# Glutamine and GABA alterations in cingulate cortex may underlie alcohol drinking in a rat model of co-occurring alcohol use disorder and schizophrenia: an 1H-MRS study

**DOI:** 10.1038/s41537-022-00272-6

**Published:** 2022-08-23

**Authors:** Patrick McCunn, Xi Chen, Barjor Gimi, Alan I. Green, Jibran Y. Khokhar

**Affiliations:** 1 Department of Anatomy and Cell Biology, Schulich School of Medicine and Dentistry, University of Western Ontario, London, ON Canada; 2 Department of Radiology, Biomedical NMR Research Center, Geisel School of Medicine at Dartmouth, Lebanon, NH USA; 3 Psychotic Disorders Division, McLean Hospital, Belmont, MA USA; 4 Department of Psychiatry, Harvard Medical School, Boston, MA USA; 5 Department of Psychiatry, Geisel School of Medicine at Dartmouth, Lebanon, NH USA

**Keywords:** Schizophrenia, Schizophrenia

## Abstract

Alcohol use disorder commonly occurs in patients with schizophrenia and significantly worsens the clinical course of the disorder. The neurobiological underpinnings of alcohol drinking are not well understood. Magnetic resonance spectroscopy (MRS) has been used to assess the neurochemical substrates that may be associated with alcohol drinking in patients; however, the causal impact of these findings remains elusive, highlighting the need for studies in animal models. This study performed MRS in the neonatal ventral hippocampal lesioned (NVHL) rat model, a model of co-occurring schizophrenia and substance use disorders. NVHL lesions (or sham surgeries) were performed on post-natal day 7 and animals were given brief exposure to alcohol during adolescence (10% v/v in a 2-bottle choice design). Animals were re-exposed to alcohol during adulthood (20% v/v) until a stable drinking baseline was established, and then forced into abstinence to control for the effects of differential alcohol drinking. Animals were scanned for MRS after one month of abstinence. NVHL rats consumed significantly more alcohol than sham rats and in the cingulate cortex showed significantly higher levels of GABA and glutamine. Significantly lower GABA levels were observed in the nucleus accumbens. No differences between the NVHL and sham animals were observed in the hippocampus. Correlation analysis revealed that GABA and glutamine concentrations in the cingulate cortex significantly correlated with the rats’ alcohol drinking prior to 30 days of forced abstinence. These findings suggest that a potential dysfunction in the glutamate/GABA–glutamine cycle may contribute to alcohol drinking in a rat model of schizophrenia, and this dysfunction could be targeted in future treatment-focused studies.

## Introduction

Alcohol use disorders (AUD) commonly occur in patients with schizophrenia (43–65%)^1^; this use significantly worsens the course of the disorder contributing to poor treatment response, treatment non-compliance^2^, relapse^3,4^, violence^5,6^, and suicide^7,8^. Unfortunately, both the current understanding of, and treatment options for, co-occurring alcohol use in patients with schizophrenia are limited. The most effective current treatment, the atypical antipsychotic clozapine, is rarely used due to its side-effect profile^9^, underscoring the need to new treatment development and investigation of potential therapeutic targets.

While the self-medication hypothesis is often used to explain alcohol and other substance use in patients with schizophrenia, we, and others, have advanced an alternative unifying hypothesis that suggests that co-occurring substance use may be related to shared genetic susceptibility or brain reward circuit dysfunction, that makes these patients especially vulnerable to the effects of substances^10–12^. Altered glutamatergic neurotransmission in particular has been heavily implicated in both schizophrenia and alcohol use disorder and may provide a potential mechanistic link to the neurobiological underpinnings of the increased predisposition to alcohol drinking^13^. Previous work has suggested that alterations in glutamine-glutamate metabolism may in fact predate alcohol abuse^14^, leading to the question of whether schizophrenia-induced glutamatergic dysfunction may be a contributor to AUD. However, glutamatergic neurotransmission remains difficult to measure in-vivo, thereby making it difficult to quantify dysfunction in the glutamate/GABA–glutamine cycle^15^.

Proton magnetic resonance spectroscopy (MRS) is a useful methodology that is often used to study the neurochemical correlates of psychiatric illness. Particularly relevant to this study, MRS allows for the assessment of glutamatergic function and dysfunction via the measurement of in-vivo concentrations of amino acid-based neurotransmitters and their metabolites (i.e., GABA, glutamine, and glutamate) within specific regions of the brain specified by three-dimensional spectroscopy voxels. Employing this technique in rodent models can allow for replication into animal models of metabolite concentration changes reported in MRS studies of human patients for further validation of the models, to establish causality, to identify therapeutic targets, and to use MRS measurements as outcome measures in treatment development.

Compounding the difficulty of detecting subtle neurobiological alterations, is the difficulty in developing accurate heuristic models in animals that adequately capture the features of clinical presentation in humans; this process becomes particularly difficult when modeling co-occurring disorders such as schizophrenia and AUD^16^. To model “dual-diagnosis” in patients, we employed an established animal model of schizophrenia and co-occurring alcohol use disorder built upon the neonatal ventral hippocampal lesioned (NVHL) rat^17–19^, which is one of the best-described animal models of SCZ^20^. This animal (experimentally created by producing an excitotoxic lesion in the ventral hippocampus in a 7-day old rat) has strong construct, face, and predictive validity for schizophrenia^21^, and displays behavioral and neurobiological dysfunctions analogous to those seen in patients with schizophrenia. Moreover, like patients with schizophrenia^1^, NVHL rats display enhanced sensitivity to, and increased use of, alcohol^22,23^, cocaine^24–26^, nicotine^27^, ketamine^28^, and methamphetamine^29^ as well as altered rewarding effects of cannabinoids^30,31^. It has been shown that this model shows consistent circuit dysfunctions with those observed in patients with schizophrenia, and that cannabis vapor exposure can worsen these dysfunctions^19,32^. We have recently shown that the NVHL rat model of schizophrenia and alcohol use disorder, initially described by ref. ^22^, can be used to understand the behavioral and neurobiological underpinnings of this disorder, specifically showing that impairments in switching behaviors (as measured by a latent inhibition of autoshaping task) contribute to the risk for alcohol drinking in this model^17^.

In this study, we utilized MRS in the cingulate cortex, nucleus accumbens, and hippocampus to assess the relationship between glutamatergic dysfunction and alcohol drinking in a rat model of co-occurring AUD and schizophrenia. Glutamine and glutamate levels are known to normalize to baseline levels by 3-weeks after last alcohol exposure^33^, therefore NVHL rats with established alcohol drinking were forced into abstinence for a month. We aimed to replicate and extend previous findings from abstinent patients with a history of alcohol use disorder^14^ while ensuring that the differential levels of drinking between the NVHL and sham rats did not confound our findings. Further, based on these previous findings^14^, we hypothesized that glutamatergic dysfunction would correlate with alcohol consumption prior to 30-day abstinence, supporting a potential contributing effect of this dysfunction to alcohol drinking in our model, and by extension to AUD in patients with schizophrenia.

## Methods and materials

### Subjects

Timed pregnant Sprague-Dawley dams were ordered from Charles River (Wilmington, MA) to arrive at gestational day 13 and were singly housed with ad libitum access to food and water. Male rat pups used in the study were individually housed in a colony room maintained on a 14:10 h light-dark cycle. Experimentation took place during the light period of the cycle. Rats were monitored and cared for in compliance with the Association for Assessment and Accreditation of Laboratory Care guidelines and the IACUC of Dartmouth College.

### NVHL preparation and surgery

Male Sprague-Dawley rat pups (*n* = 11) on post-natal day 7 (PND 7, 15–20 g) were anesthetized using hypothermia and placed on a Styrofoam platform attached to a stereotactic apparatus (Kopf Instruments, Tujunga). For the NVHL group, pups (*n* = 6) were bilaterally injected with excitotoxic ibotenic acid (3.0 μg ibotenic acid [Tocris, Minneapolis] dissolved in 0.3 μl of artificial cerebrospinal fluid (aCSF; 150 μM Na, 3.0 μM K, 1.4 μM, Ca, 0.8 μM Mg, 1.0 μM P, and 155 μM Cl; pH 7.4)^34^ into their ventral hippocampi (AP −3.0 mm, ML ± 3.5 mm, VD ± 5.0 mm relative to bregma). The remaining pups (*n* = 5) were injected with aCSF at the same coordinates (Sham, unlesioned). After the surgery, wounds were closed using tissue glue, and rats were returned to their dams when activity level had returned to normal. On PND21, rats were weaned and housed individually for the duration of the study to allow for individual measurement of alcohol drinking.

### Alcohol drinking in adolescence and adulthood

To ensure that the NVHL rats would be susceptible to drinking alcohol preferentially in adulthood, we followed the protocol of Jeanblanc et al.^22^, in which the rats were given access to alcohol in a free-access 2-bottle (water and 10% alcohol v/v in spill-proof bottles) design between PND 28 and 42. At the end of this period, the alcohol bottle was removed, and the rats only had access to water until they reached adulthood. Water, alcohol, and food intake as well as body weight were measured daily during PND 28–42 and then again in adulthood upon resumption of alcohol drinking. Alcohol was then reintroduced to the rats in adulthood (PND 90) in a continuous-access 2-bottle choice (water and 20% alcohol v/v) design. The two bottles were rotated daily to prevent positional preference, consistent with our previous investigations^35^. Animals were allowed to drink until their drinking stabilized (4 days with less than 10% variance in drinking) and were allowed to drink at these levels for 30 days, followed by forced abstinence. Since glutamine and glutamate levels normalize to baseline levels by 3-weeks after last alcohol exposure^33^, we chose to scan the animals 30 days after the last alcohol drinking day.

### MRI methods

All MRI scans were performed on an Agilent 9.4T animal scanner using VnmrJ 4.0A software located at the Dartmouth-Hitchcock Medical Center, with a RAPID 2 channel volume coil as a transmitter and a 4-channel phase-array surface coil as receiver. Rats were anesthetized with an isoflurane-oxygen mixture (5% for induction and 2.0–2.5% for maintenance) and the respiration rate was maintained between 40 and 60 respiration per minute. The rectal temperature was kept at 36.5 °C using a blown hot air system. The sagittal/axial anatomic images were acquired using a multiple spin-echo sequence (TR/TE = 2000/12 ms, echo number = 8, average = 4) with the anterior commissure used as a landmark for axial image positioning. Imaging voxel size was 0.16 × 0.16 × 1 mm^3^.

MRS data were acquired from the cingulate cortex (1.8 × 1.6 × 3.6 mm^3^), dorsal hippocampus (1.8 × 0.8 × 2.6 mm^3^) and nucleus accumbens (1.6 × 1.8 × 2.4 mm^3^) using the spectroscopic voxels listed. For all acquisitions, an optimized short TE PRESS sequence (TE/TR = 14/2000 ms) was used. The spectroscopic voxel sizes and precise locations of placement were chosen according to ref. ^36^ and inclusion of CSF and white matter in the voxels was minimized. 3D shimming was performed before each MRS acquisition such that the linewidth of the water from each spectroscopic voxel was <14 Hz. The number of averages used, and acquisition time were 768 (28 min), 1024 (38 min), and 1536 (56 min) for the cingulate cortex, hippocampus, and nucleus accumbens, respectively. To minimize chemical shift displacement error for neurotransmitters of interest (Glu, Gln, GABA), the center frequencies of the localization radiofrequency pulses of the PRESS sequence were set to 2.3 ppm. Water suppression was performed using the variable pulse power and optimized relaxation delays water suppression (VAPOR) scheme^37^ using Gaussian-shaped suppression pulses with a bandwidth of 200 Hz, prior to acquisition of water-suppressed MRS data. For each region, unsuppressed water signal was also acquired using the same sequence, except with the RF pulses turned off during the VAPOR scheme. For acquisition of the water signal, the RF pulses were centered on the water signal at 4.7 ppm. Data were acquired in a metabolite-water interleaved fashion, with 16 averages of unsuppressed water signal acquired between 8-min blocks of water-suppressed acquisition (256 averages).

Preprocessing of the MRS data was performed in the console software VnmrJ 4.0 with macros written in-house. Signals from different coil channels were combined, and phase and frequency corrections of water-suppressed metabolite spectra were done. Since acquisition was done in an interleaved fashion, metabolite spectra acquired during each 8-minute block were phase and frequency corrected using the water spectrum acquired afterward as a reference, for the slow drifts caused by the magnet and potential animal movements during the long acquisition time. Processing of acquired metabolite spectra, spectral fitting, and metabolite quantification was performed with LCModel^38^ version 3.6-1H. A basis set consisting of simulated metabolite spectra produced in VeSPA^39^ with the same acquisition parameters and an experimentally acquired macromolecule spectrum were provided to LCModel for spectral fitting. The macromolecule spectrum was acquired from a whole brain spectroscopic voxel and was included in the basis set to account for the macromolecule baseline present in the spectra due to the short TE used. The unsuppressed water signal was provided to LCModel for both the eddy current correction as well as the reference signal for metabolite concentration calculation.

### Lesion verification and analysis

After euthanasia, the rat brains were removed and flash frozen. Lesions were verified via 40 mm sections of the dorsal and ventral hippocampus using a freezing microtome, that were mounted on glass slides and thionin stained. The hippocampus was examined for bilateral damage microscopically (typically includes cell loss, thinning, cellular disorganization, and ventricular enlargement).

### Data analysis

An unpaired Student’s *t*-test was applied to compare alcohol consumption between the Sham and NVHL groups and neurometabolite concentration between the Sham and NVHL groups. When statistically significant differences were detected in neurometabolite concentrations a Pearson correlation analysis was used to determine the correlation between alcohol drinking and neurometabolite concentration. For all statistical analysis, *p* < 0.05 was taken as statistically significant. The data that supports the findings of this study is available from OSF at https://osf.io/d8xrs/.

## Results

### Histology

**Fig. 1:**
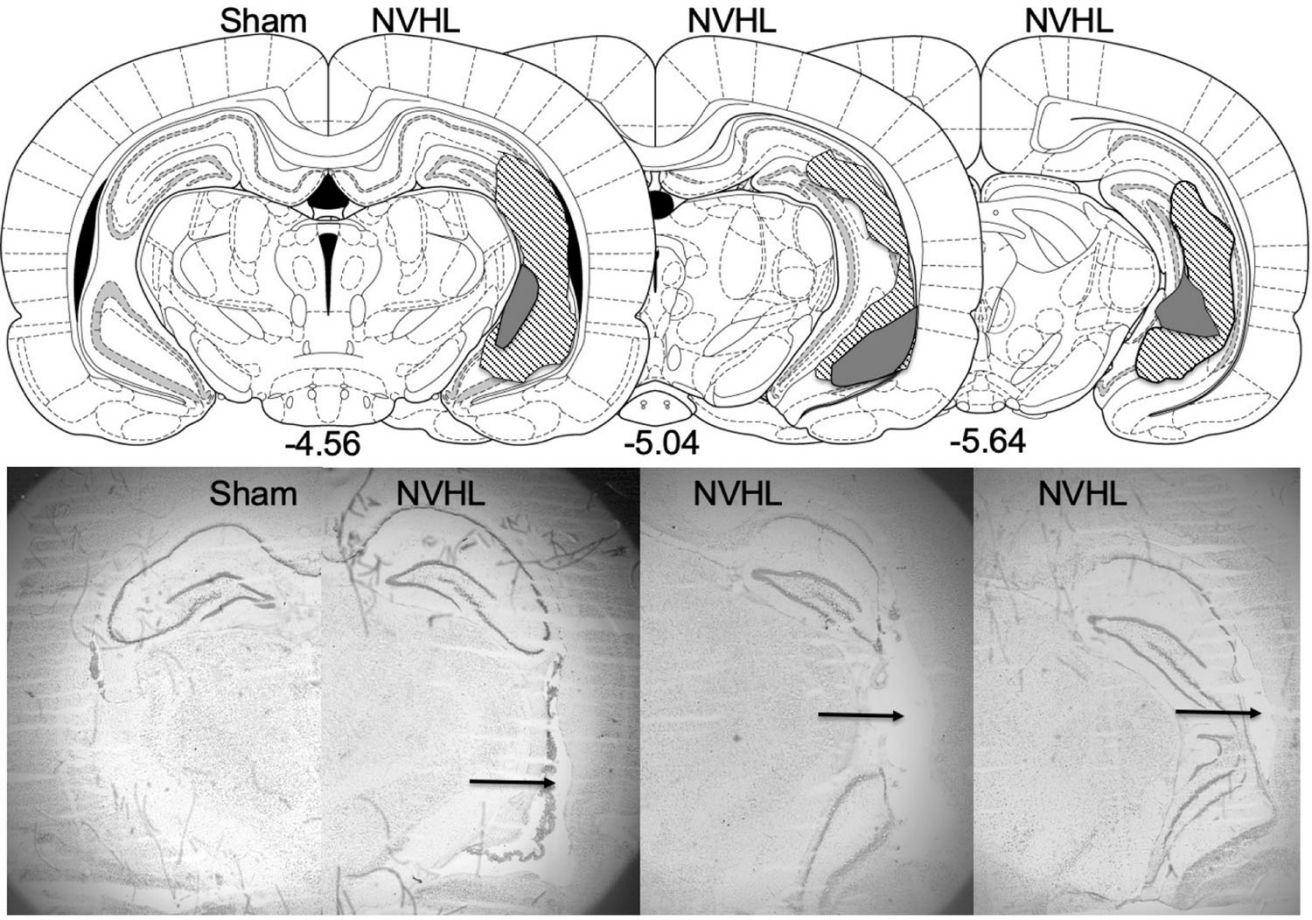
Schematic and representative photomicrographs depicting lesion extent and location in NVHL rats. Arrows point to a lesion in a representative NVHL rat’s ventral hippocampus photomicrographs (right) compared to a sham rat (left).

All NVHL rats displayed bilateral lesions of the hippocampus, whereas no lesions were observed in the sham animals. Representative photomicrographs depicting lesion location and extent are presented in Fig. 1.

### MRS

**Fig. 2:**
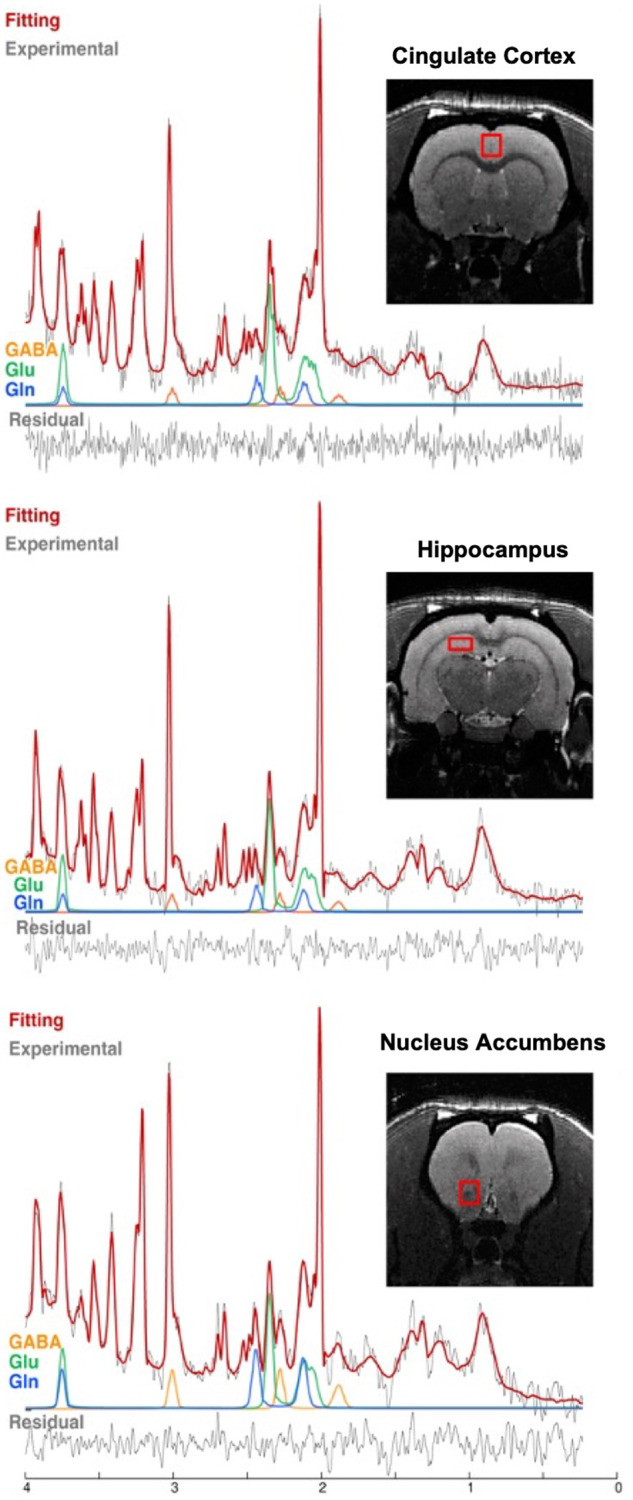
LCModel fitting and voxel positions. LCModel fittings (red line) of the experimental spectra (black line) of the cingulate cortex (top), hippocampus (middle), and nucleus accumbens (bottom) of representative NVHL and sham rats, with inset showing the voxel position.

Figure 2 shows a representative LCModel fitting of spectra in the cingulate cortex, hippocampus, and nucleus accumbens of NVHL and sham rats with the spectroscopic voxel marked on axial/sagittal images. Quality control data including the linewidth, SNR, and the Cramer-Rao lower bound are summarized in Table 1.

**Table 1:** Cramer-Rao lower bound (CRLB), Linewidth (Hz), and Signal to Noise Ratio (SNR) of the cingulate cortex, hippocampus, and nucleus accumbens of all spectra.

	CRLB			Linewidth (Hz)	SNR
	GABA	Glu	Gln		
Cingulate Cortex	23 ± 7%	4.0 ± 0.3%	15 ± 5%	9 ± 2	18 ± 4
Hippocampus	28 ± 8%	7 ± 2%	24 ± 9%	8 ± 2	14 ± 2
Nucleus Accumbens	26 ± 6%	7 ± 1%	18 ± 8%	10 ± 2	9 ± 4

### Alcohol consumption

**Fig. 3:**
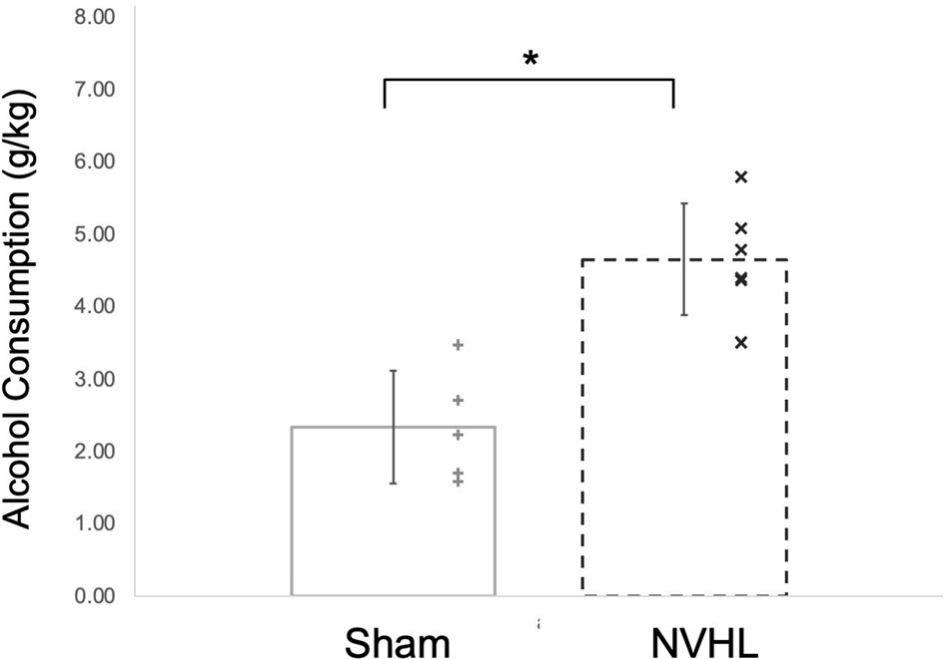
Higher levels of alcohol drinking in NVHL rats compared to sham rats. NVHL rats drank significantly more alcohol than the sham group measured as an average of the last 3 days before withdrawal (sham = 2.92 ± 0.78 g/kg, NVHL = 4.65 ± 0.77 g/kg). Statistically significant differences are denoted by * (*p* < 0.001; *n* = 6 NVHL, 5 sham). Data are shown as mean ± SD. Individual data points are denoted with a (+) Sham or (x) NVHL.

Alcohol consumption was measured as an average of the last three days before forced abstinence in both the sham and NVHL groups. Consistent with our previous findings, results of the t-test revealed NVHL rats drank significantly more alcohol than the sham group (NVHL = 4.65 ± 0.77 g/kg, Sham = 2.92 ± 0.78 g/kg, *p* < 0.001, Fig. 3). No differences in alcohol drinking were observed between NVHL and sham rats during adolescence.

### Neurometabolites

**Fig. 4:**
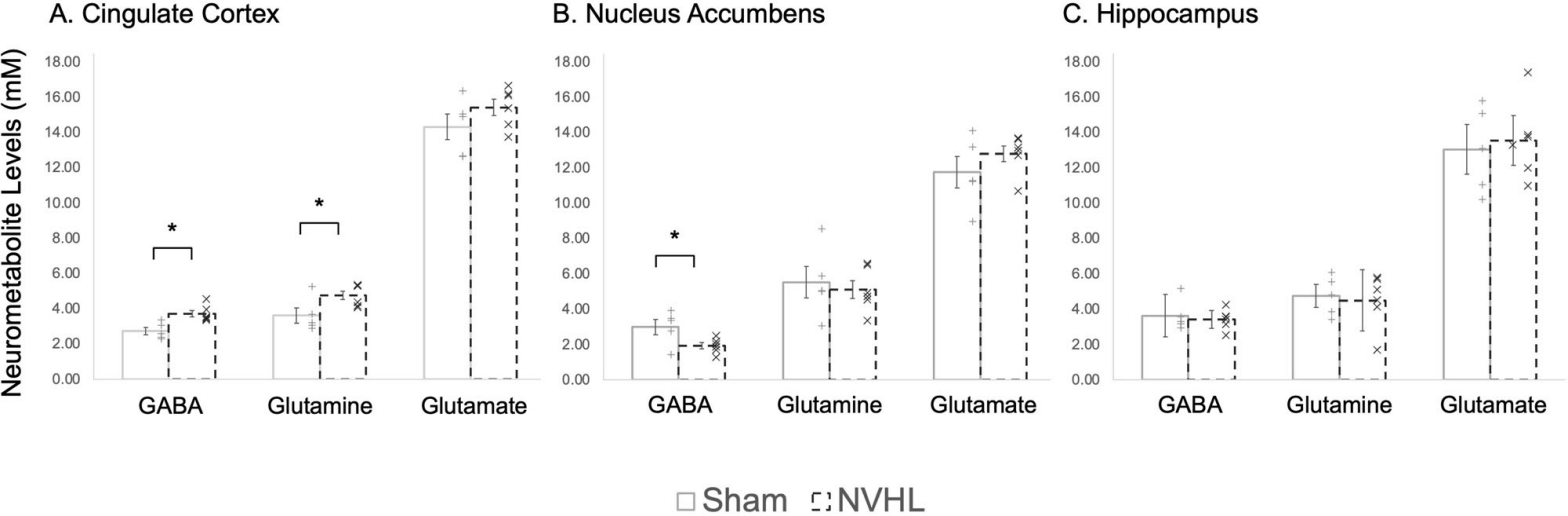
Higher cingulate glutamine and GABA, but lower nucleus accumbens GABA, levels in NVHL rats. In the cingulate cortex (**A**), GABA and glutamine concentrations were significantly higher in the NVHL rat compared to the sham rats. In the nucleus accumbens (**B**) of the NVHL group, GABA concentrations were significantly lower than the sham group. No differences between groups were observed in the dorsal hippocampus (**C**). Statistically significant differences between groups are represented with * (*p* < 0.05; *n* = 6 NVHL, 5 sham). Data are shown as mean ± SD. Individual data points are denoted with a (+) Sham or (x) NVHL.

In the cingulate cortex of the NVHL group, a t-test revealed both GABA and Gln concentrations were statistically significantly higher than the sham group (GABA: *p* = 0.0061, Cohen’s *d*: 5.0365; Gln: *p* = 0.0412, Cohen’s *d*: 3.1967), while Glu showed no statistically significant difference. In the nucleus accumbens of the NVHL group only GABA concentrations were statistically significantly lower than the sham group (*p* = 0.0367, Cohen’s *d*: 3.2347), while Gln and Glu showed no statistically significant difference. There were no statistically significant differences in concentrations of the three metabolites in the hippocampus. Table 2 and Fig. 4 provide the complement of results of metabolites in all three brain regions in both groups.

**Table 2:** Average concentrations (mM ± Standard Deviation) of GABA, glutamine (Gln), and glutamate (Glu) in the cingulate cortex, hippocampus, and nucleus accumbens of NVHL and sham rats.

Averaged Concentrations (mM ± Standard Deviation) for Neurometabolites			
	GABA	Glutamine	Glutamate
Cingulate Cortex			
Sham	2.7262 ± 0.2033	3.6062 ± 0.4337	14.3130 ± 0.7289
NVHL	3.7113 ± 0.1875	4.7500 ± 0.2520	15.4058 ± 0.4597
*p* value	0.0061*	0.0412*	0.2212
Nucleus Accumbens			
Sham	2.9850 ± 0.4319	5.5076 ± 0.8892	11.7468 ± 0.8883
NVHL	1.9210 ± 0.1728	5.0982 ± 0.5079	12.7987 ± 0.4506
*p* value	0.0367*	0.6858	0.2940
Hippocampus			
Sham	3.6268 ± 1.1933	4.7470 ± 0.6458	13.0428 ± 1.3991
NVHL	3.4128 ± 0.4944	4.4908 ± 1.7322	13.5497 ± 1.3989
*p* value	0.6385	0.2722	0.1184

Values showing statistically significant differences (*p* < 0.05) between groups are bolded and represented with *. Data are shown as mean ± SD.

### Correlation analysis

**Fig. 5:**
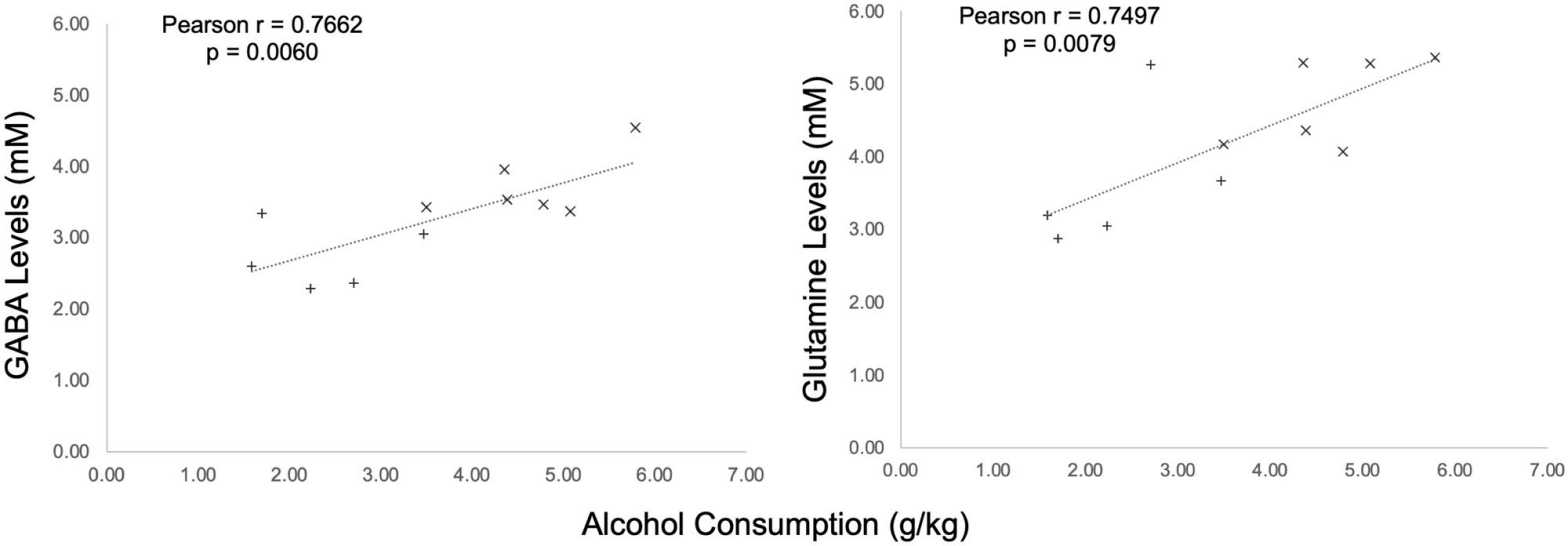
Glutamine and GABA levels in the cingulate cortex correlate with alcohol drinking prior to abstinence. Pearson correlation between (left) alcohol consumption (g/kg) and GABA (mM), and (right) alcohol consumption (g/kg) and glutamine concentration (mM) in the cingulate cortex. Alcohol drinking showed a positive correlation between both GABA (*r*(9) = 0.7662, *p* = 0.0060) and glutamine concentration (*r*(9) = 0.7497, *p* = 0.0079) as measured by magnetic resonance spectroscopy (MRS) (*n* = 6 NVHL, 5 sham). Individual data points are denoted with a (+) Sham or (x) NVHL.

Performing a Pearson correlation between alcohol drinking (taken as the average of the last 3 days of alcohol drinking prior to 30 days of abstinence) and the neurometabolites as measured by MRS, it was found that alcohol drinking showed a positive correlation to both GABA and Gln in the cingulate cortex (GABA: *r*(9) = 0.7662, *p* = 0.0060, Cohen’s *d*: 2.3847; Gln: *r*(9) = 0.7497, *p* = 0.0079, Cohen’s *d*: 2.2657). No other statistically significant correlations were found. Refer to Table 3 and Fig. 5 for full results.

**Table 3:** Pearson Correlation analysis of alcohol consumption (g/kg) and neurometabolites (mM).

	Alcohol and GABA	Alcohol and Glutamine	Alcohol and Glutamate
Cingulate Cortex			
Pearson *r*	0.7662	0.7497	0.3919
*t*	3.5769	3.3987	1.2779
*p* value	0.0060*	0.0079*	0.2332
Nucleus Accumbens			
Pearson *r*	−0.3599	−0.2121	0.3206
*t*	1.1576	0.6510	1.015
*p* value	0.2768	0.5313	0.3364
Hippocampus			
Pearson *r*	−0.0495	−0.0956	−0.2204
*t*	0.1486	0.2881	0.6780
*p* value	0.8852	0.7798	0.5148

Alcohol drinking showed a positive correlation to both GABA (*r*(9) = 0.7662, *p* = 0.0060) and Glutamine concentration (*r*(9) = 0.7497, *p* = 0.0079) as measured by Magnetic Resonance Spectroscopy (MRS) (*n* = 6 NVHL, 5 sham).

Values showing statistically significant differences (*p* < 0.05) between groups are bolded and represented with *.

## Discussion

This study utilized MRS to assess the relationship between glutamatergic dysfunction and alcohol drinking in a neurodevelopmental rat model of co-occurring AUD and schizophrenia. Consistent with our previous findings, the NVHL rats showed significantly higher alcohol consumption (who then underwent 30 days of abstinence prior to being scanned for neurometabolite levels). Proton MRS in the cingulate cortex revealed significantly higher levels of GABA and glutamine while significantly lower GABA levels were also observed in the nucleus accumbens. No differences in neurometabolite levels in the hippocampus were observed between the NVHL and sham animals. In the cingulate cortex both GABA and glutamine showed a positive correlation with the amount of alcohol consumed prior to 30 days of forced abstinence. No statistically significant changes were observed in the hippocampus, despite being the site of the lesions; it should be noted though that MRS was collected in the dorsal hippocampus. These findings provide support that glutamatergic dysfunction may be related to alcohol drinking and may contribute to co-occurring alcohol use disorder in patients with schizophrenia.

While the neurometabolic correlates of dual-diagnosis schizophrenia and alcohol have not been studied explicitly, glutamatergic dysfunction is known to occur in both schizophrenia alone and in AUD alone. The increased glutamine observed in the cingulate cortex in our study is consistent with previous reports in pharmacological animal models of schizophrenia^40^ and in patients with schizophrenia^41,42^. Our findings of the elevated GABA in the same region are in line with a recent report on chronic schizophrenia^43^, while the reduced GABA levels in the nucleus accumbens of decreased GABA are also consistent with post-mortem findings from patients with schizophrenia^44^, consistent with decreases in glutamic acid decarboxylase 67 (GAD67, the enzyme that converts glutamate to GABA) mRNA in the nucleus accumbens in patients with schizophrenia^45^. Further, the increase in glutamine in the cingulate cortex has also been observed in human subjects with current AUD and those in remission^14^. Together, these findings indicate that the dysfunction of the glutamate/GABA–glutamine cycle observed in the NVHL model appears to be similar to the dysfunctions seen in patients with schizophrenia and/or AUD alone. Interestingly, no differences in neurometabolite levels in the dorsal hippocampus were observed. These findings contrast previous studies which suggest lower GAD67 expression in the dorsal hippocampus^46^; however, these findings were specific to the CA1 region suggesting that the dorsal hippocampus voxel may not have captured the sub-region-related changes. Importantly, to our knowledge, none of the MRS studies of patients with schizophrenia have focused on the posterior hippocampus (dorsal hippocampus in primates)^47^. These findings lend some support for the NVHL model as a potentially useful heuristic studying schizophrenia and alcohol use disorder, which we can now begin to manipulate pharmacologically toward treatment development. However, it is possible that the neurometabolic correlates of the co-occurring disorder are distinct from those of the two disorders alone, thereby supporting the need for more studies in dual diagnosis patients to gain a better understanding of the mechanisms contributing to the development of AUD in patients with schizophrenia.

Previous work has shown that glutamine and glutamate levels normalize to baseline levels by 3-weeks after last alcohol exposure in the rat brain^33^. In order to control for the potential effects of the higher levels of alcohol drinking in the NVHL rats, rats in this study were forced into abstinence for 30 days prior to MRS acquisition; therefore, the higher glutamine levels are unlikely to be due to the effects of higher alcohol consumption or withdrawal from alcohol drinking. The presence of the higher glutamine levels even after these 30 days, and the correlation between GABA and glutamine levels and past drinking, also supports previous hypotheses from studies in patients with AUD^14^ that glutamatergic dysfunction may predate alcohol exposure and could contribute to the vulnerability for AUD in patients (with or without schizophrenia). Another recent study in patients with AUD undergoing monitored abstinence found prospective associations between anterior cingulate glutamine levels (but not GABA which was found to be significantly increased) and heavy drinking, craving and withdrawal symptoms^48^. This is further supported by MRS studies in substance-naïve adolescents with a family history of AUD, where the glutamine to glutamate ratio correlated with impulsive behaviors, suggesting a role for glutamatergic dysfunction in the vulnerability toward alcohol use^49^. Moreover, craving during detoxification in alcohol-dependent individuals is also related to combined glutamine-glutamate levels in the cingulate cortex^50^. However, it is still possible that the higher levels of alcohol drinking and withdrawal in the NVHL rats contributed to increases in the packing density of glutamine synthetase expressing astrocytes^51^, which might have contributed to the greater conversion of glutamate to glutamine. Alternatively, since astrocytic genes are often upregulated in brains from patients with schizophrenia^52^, it is possible that the higher levels of glutamine are related to such upregulation in the NVHL rat, and not due to alcohol drinking. Future studies will assess neurometabolite levels prior to initiation of alcohol drinking to assess the potential predictive potential of cingulate cortex glutamine levels on alcohol drinking, while also uncovering what roles alcohol drinking and astrocyte expression might contribute to the glutamatergic dysfunction observed here.

The current study does have some limitations, the most important of which is the lack of sham and NVHL groups with no alcohol exposure during adolescence. This limits our ability to assess the differential effect of adolescent alcohol exposure as it relates to the NVHL model (aside from its known impact on inducing alcohol drinking in adulthood). It could be the NVHL surgery, the alcohol drinking during adolescence, or the interaction between these factors, has led to the glutamatergic dysfunction observed in the current study. Furthermore, the limited sample sizes do not permit robust correlations using only the NVHL rats, which might have been useful in understanding whether the differences in neurometabolite levels relate just to the differences in alcohol drinking between NVHL or sham animals (both of which drink different amounts of alcohol) or are related to other factors that may contribute to this dysfunction (e.g., NVHL lesion). Therefore, these results must be interpreted with caution. Follow up studies will attempt to determine the nature of impact of adolescent alcohol drinking on the neurometabolic dysfunctions in NVHL rats utilizing a group without adolescent alcohol exposure.

Abnormal glutamatergic neurotransmission or metabolism has been previously linked to both schizophrenia and AUD, but also to other psychiatric disorders such as bipolar disorder and major depressive disorder, making it a potentially valuable trans-diagnostic target for treatment development^15^. With growing evidence of glutamat-/GABA-ergic dysfunction predating AUD in patients (with or without schizophrenia), more targeted treatment options tackling this dysfunction may be explored to limit the detrimental effect alcohol consumption may have on these patients.

## Conclusion

Consistent with previous studies in patients with schizophrenia, glutamatergic dysfunction was observed in the cingulate cortex of NVHL rats. NVHL rats consumed significantly more alcohol than sham rats and had significantly higher levels of GABA and glutamine in the cingulate cortex as well as significantly lower levels of GABA in the nucleus accumbens. GABA and glutamine showed a positive correlation with alcohol consumption across all animals (measured as the last 3 days of alcohol drinking prior to 30 days abstinence). These findings provide additional support for the hypothesis that glutamat-/GABA-ergic dysfunctions may contribute to the vulnerability for alcohol drinking, while providing a potential avenue where schizophrenia-induced neurometabolic dysfunctions may give rise to a shared susceptibility for both schizophrenia and the development of AUD.
